# Design and Calibration of a New 6 DOF Haptic Device

**DOI:** 10.3390/s151229857

**Published:** 2015-12-11

**Authors:** Huanhuan Qin, Aiguo Song, Yuqing Liu, Guohua Jiang, Bohe Zhou

**Affiliations:** 1 School of Instrument Science and Engineering, Southeast University, Nanjing 210096, China; 230159227@seu.edu.cn; 2 National Key Laboratory of Human Factors Engineering, China Astronaut Research and Training Center, Beijing 100094, China; clara_liu@163.com (Y.L.) ; jgh_isme@sina.com (G.J.) ; zhoubohe@126.com (B.Z.)

**Keywords:** haptic device, force feedback, position tracking, hybrid structure, calibration

## Abstract

For many applications such as tele-operational robots and interactions with virtual environments, it is better to have performance with force feedback than without. Haptic devices are force reflecting interfaces. They can also track human hand positions simultaneously. A new 6 DOF (degree-of-freedom) haptic device was designed and calibrated in this study. It mainly contains a double parallel linkage, a rhombus linkage, a rotating mechanical structure and a grasping interface. Benefited from the unique design, it is a hybrid structure device with a large workspace and high output capability. Therefore, it is capable of multi-finger interactions. Moreover, with an adjustable base, operators can change different postures without interrupting haptic tasks. To investigate the performance regarding position tracking accuracy and static output forces, we conducted experiments on a three-dimensional electric sliding platform and a digital force gauge, respectively. Displacement errors and force errors are calculated and analyzed. To identify the capability and potential of the device, four application examples were programmed.

## 1. Introduction

Traditional interaction research emphasizes visual and auditory display, but pay little attention to haptic display. Visual and auditory interactions use vision and voices to transmit information. These interactions are non-contact interaction approaches, and they have some limitations. For example, vision mainly dominates visual percepts like size, shape and position [1], but kinematic feelings such as velocity, acceleration or inertia cannot be reflected, even though vision has a higher spatial resolution than touch. Haptic refers to touching or interacting with real, virtual and remote environments, such as exploring and distinguishing material properties [2,3] and tele-operating a robot. Different from visual and auditory display, haptic is a unique bilateral sensory modality with energy and information flowing both from and to the user. This bi-directionality is often referred to as the single most important feature of the haptic interaction modality [4]. As a medium between environments and human users, haptic interfaces transmit and display haptic stimuli [5]. With precisely controlled forces and torques exerted on the manipulator’s fingertips, hand or arm, subtle sensations are able to be perceived, thus a high level of immersion is constructed. With the development of computer science, haptic has manifested great superiorities in fields, ranging from robotics and tele-operation to computational geometry and computer graphics, and to psychophysics, cognitive science and the neurosciences [4]. Nowadays, haptic interfaces have been widely applied in many areas such as education [6], entertainment [7], surgical simulation and training [8], and scientific visualization [9].

The past decade has shown an increasing interest in the science of haptics. A number of haptic devices with different structures have been developed, some of which are commercially available devices and some are experimental prototypes. The PHANToM haptic device (SensAble Technologies, Woburn, MA, USA) designed by Massie and Salisbury [10,11] is a convenient desktop device with 3 or 6 actuated DOF. Due to characteristics of low inertia, low friction and high position precision, it has been widely used in a multitude of applications. Although it shows great success, there are still some weaknesses, such as limited strength, low stiffness and small workspace, *etc.* A critical study of the mechanical and electrical properties of the PHANToM is presented in [12]. After the research of the kinematics, dynamics, high frequency dynamic response, and velocity estimation of the PHANToM system, some modifications are made to compensate for the deficiencies that impede high performance. The Novint Falcon is a relatively inexpensive 3 DOF haptic device made by Novint for the gaming industry [13]. It has features of low inertia, high stiffness, high operating rate and better position repeatability due to the adoption of the DELTA mechanism [14]. Nevertheless, it still has some shortages. Firstly, its workspace is small, and output forces are relatively small and inaccurate. Secondly, complexity of kinematic modelling increases significantly compared to its serial counterparts. In fact, some of the same advantages and disadvantages exist in haptic devices such as the DELTA Haptic Device (Force Dimension, Nyon, Switzerland) [15], OMEGA Haptic Devices (Force Dimension, Nyon, Switzerland), haptic prototypes designed by Jumpei Arata *et al.* [16] and Minh Hung Vu *et al.* [17]. The VISHARD6 [18] and VISHARD10 [19] are both haptic devices with a serial kinematic design. The VISHARD6 is a 6 DOF device designed towards a comparatively large workspace and high force capability. However, it suffers from low mechanical stiffness. Singularities exist in its workspace as well. The VISHARD10 with 10 DOF introduces actuated kinematic redundancies to realize a larger workspace free of singularities while simultaneously reduce the device size. However, serial structure may lead to large occupation in space and loss in portability. On these issues, both devices do not have any breakthroughs. The same disadvantages are reflected in haptic devices such as HapticMaster [20].

Traditionally, structure of haptic devices can be divided into serial and parallel structures. The serial structure is an open kinematic structure, which usually provides a large workspace, but shows lack of strength. Furthermore, singularities may exist in the workspace, whereas the parallel structure has a higher level of stiffness and better position repeatability. However, applications have been limited by its small workspace and complex kinematic modelling. Consequently, a hybrid structure that combines advantages of both structures is proposed. This form has proven itself as an excellent platform of large workspace, high output ability and high stiffness. The compact 6 DOF haptic interface designed by Y.Tsumaki *et al.* [21] is such a hybrid structure device. 

With the diversification of haptic applications, many tasks need to simulate arbitrary object-object interactions. A 6 DOF haptic device that provides torque feedback in addition to force feedback is very useful. In 6 DOF haptic scenes, the virtual avatar is usually a wrench, a screwdriver or something like a teapot [22]. It gives operators enough dexterity to feel and explore virtual objects. Additionally, in some special tasks, it is necessary to simulate grabbing actions. Although a press button can solve parts of the problem sometimes, a grasping interface like CyberForce or CyberGrasp that can represent multi-finger joints is much more desirable.

Force feedback and sensing is a key technology in a virtual training system. It guarantees the authenticity of the training in many contact operations such as load transmission and component assembly. The absence of forces destructs immersions when touching and moving objects. It is necessary to have a haptic device to integrate operators into the virtual training system. Contact operations mainly relay on human upper extremity. Currently, commercially available haptic devices provide limited kinds of force feedback for upper limbs and hands. CyberTouch only provides senses of touch through vibration. CyberGrasp only represents forces of fingers. PHANTOM, HAPTION and CyberForce only provide forces on hand. The combination of CyberForce and CyberGrasp can exert forces on finger and hand, but CyberGrasp is hard to equip, and its motor-pull transfer method has electromagnetic interference problems. So it is essential to design a haptic device that combines finger and hand together, that is easy to equip, and that can realize various operations such as grasping, pushing-and-pulling and twisting in a virtual training system. Based on these motivations, a new 6 DOF haptic device with a grasping interface is designed and fabricated. This device differs from previous haptic devices on several important points. First, the integration of finger and hand increases dexterity of this device. Second, it is a new hybrid structure device designed towards a comparatively large workspace and high force output ability. Lastly, with an adjustable base, it allows operators to change different postures without interrupting haptic tasks.

The rest of this paper is organized as follows. Section 2 proposes the mechanical design of this 6 DOF haptic device. Motion measurement and tracking, as well as realization of force feedback, are introduced in Section 3 and Section 4 respectively. Section 5 describes the control scheme of this device. Specific calibration and evaluation experiments are shown in Section 6. Experimental results and error analysis are also given in this section. Finally, conclusions are made in Section 7.

## 2. Mechanical Design

**Figure 1:**
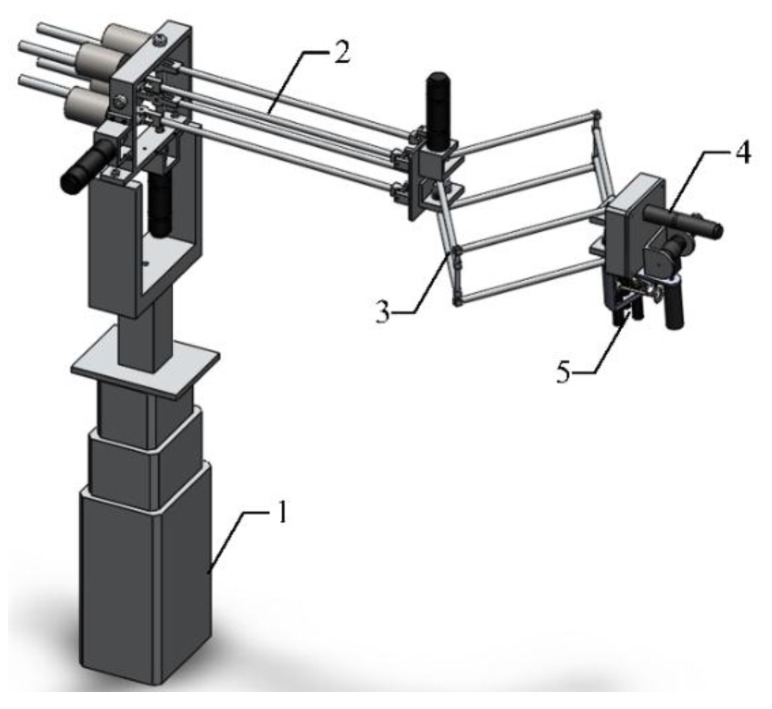
General assembly drawing (**1**) Base; (**2**) Double parallel linkage; (**3**) Rhombus linkage; (**4**) Rotating mechanical structure; (**5**) Grasping interface.

**Figure 2:**
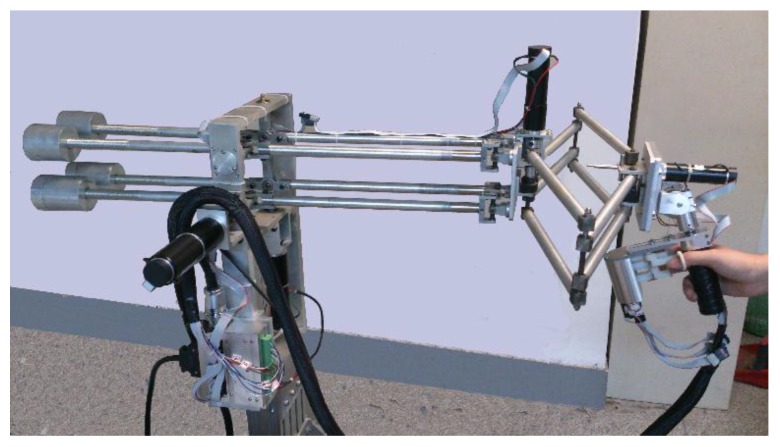
Overview of the haptic device.

Generally, an ideal haptic device is designed towards low inertial mass, low friction, low backlash, high structural stiffness, high force bandwidth and dynamic range, large workspace, and freedom of mechanical singularity [23]. However, these demands are conflicting and difficult to achieve entirely. Careful considerations should be given by designers to the selection of a variety of requirements that a desirable haptic device needs to meet. Additionally, human perceptual thresholds can be used to establish general design guidelines [24]. The main design objectives of our device are to obtain a large workspace and high output capability, and, at the same time, provide force feedback for three fingers. A hybrid structure haptic device with a grasping interface was designed. The general assembly drawing and overview of this device are presented in Figure 1 and Figure 2 individually. It is mainly composed of an adjustable base, a double parallel linkage, a rhombus linkage, a rotating mechanical structure and a grasping interface.

**Figure 3:**
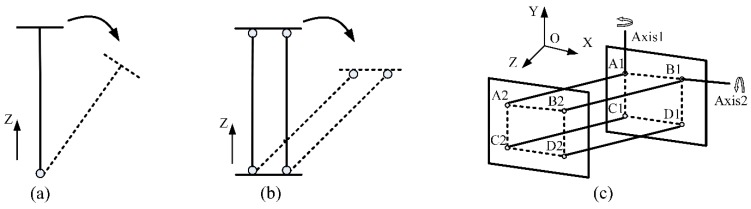
Double parallel linkage. (**a**) Single linking bar; (**b**) Two parallel linking bars; (**c**) Double parallel linkage.

The principle of the double parallel linkage is described in Figure 3. When a single linking bar rotates around a fixed point, there will be an inclination of the end-plane (see Figure 3a). Once two parallel linking bars are employed, the inclination disappears. The end-plane always keeps parallel to the initial location (see Figure 3b).

The double parallel linkage utilizes this principle. As shown in Figure 3c, there are four identical linking bars in this structure. The linking bar A1 A2, B1 B2, C1 C2 and D1 D2 are fixed on the base plane A1 B1 C1 D1 and the movable plane A2 B2 C2 D2 with universal joints at their extremities. The inclination and orientation in space of the movable plane remain unchanged, whatever motions of linking bars may be. The trajectory of the plane A2 B2 C2 D2 is a part of the spherical surface. There is only a small displacement along the Z-axis. A new structure that can realize a large independent linear motion in Z direction was designed. 

**Figure 4:**
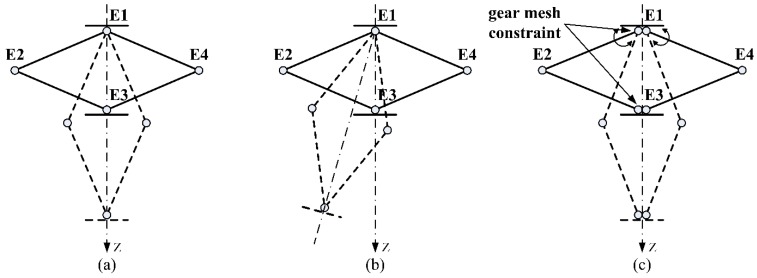
Rhombus linkage. (**a**) A large movement along the Z-axis ; (**b**) Deflection without gear mesh constraint; (**c**) Synchronous motion with gear mesh constraint.

The rhombus linkage (shown in Figure 4) is a symmetrical structure. The linking bar E1 E2 and E2 E3 are connected by an axial joint. The same connection is applied to the bar E1 E4 and E4 E3. The four linking bars constitute a deformable space-rhombus. If this structure is stretched to the dotted position, the point E3 will have a large movement along the Z-axis (see Figure 4a). But if rotation rates of the bar E1 E2 and E1 E4 are different, there will be a deflection (displayed in Figure 4b). To ensure synchronous motions, gear mesh constraints are added at the points E1 and E3 (see Figure 4c).

**Figure 5:**
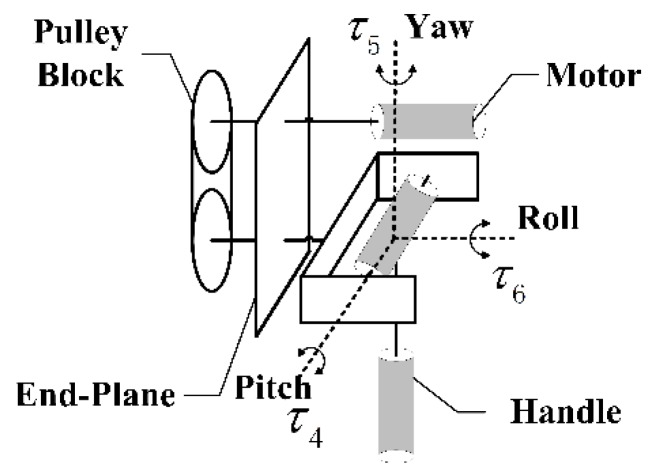
Rotating mechanical structure.

The rotating mechanical structure is shown in Figure 5. There are three motors numbered as NO. 4~NO. 6 in this structure. Each motor is perpendicular to the other two in space, so there is no coupling interference among torques. The torque τ6 generated by motor NO. 6 is transmitted to the handle through pulley block. The other two torques are transmitted through mechanisms.

**Figure 6:**
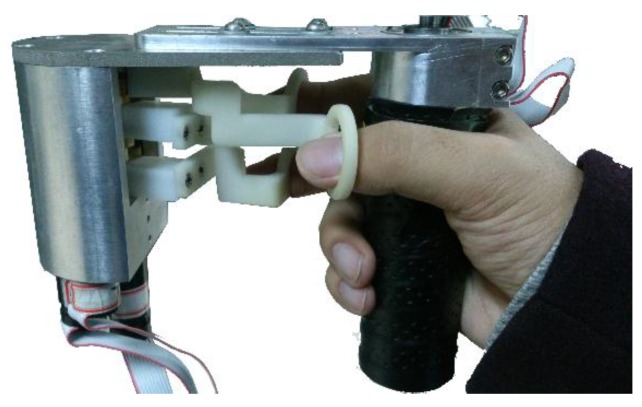
Grasping interface.

The grasping interface (shown in Figure 6) is designed to provide three independent forces to the thumb, forefinger and middle finger respectively. It is equipped with three motors and three finger rings. During operation, users are asked to hold the handle with three fingers inserted into the rings to feel grasping forces. Dynamic compensations that are based on rotation angles have been integrated to counteract the effect of gravity so that operators can feel more comfortable when grasping objects.

In addition, an adjustable base that can provide 400 mm of free adjusting space along the vertical direction is introduced to meet the requirement of different postures.

There are mainly two kinds of haptic actuators: DC motors and MR (Magneto-Rheological) actuators. Since haptic actuator is constantly working at a locked-rotor condition, it must have a high level of static performance and good cooling performance. Choosing a proper haptic actuator can enhance the haptic feedback performance dramatically, and hence provide better user experience. In this paper, the actuators chosen are Maxon DC motors, produced in Switzerland. This type of motor uses high-performance permanent magnetic steel as a magnetic component. It is a tight and efficient driving device. Because of its small polar moment of inertia, it has a short response time, which is about 2~3 ms. At the same time, Maxon also provides each motor with a supporting decelerator to reduce output rotating speed, and increases output torque and load capability. This motor is highly suitable for a haptic feedback device which has low rotating rate and large torque. The position sensor is mainly used to measure angular displacement at moveable joints. Common angular sensors include the Hall sensor, the DC tachometer, rotating transformers, and encoders. Out of these sensors, the encoder is the most commonly used angular sensor. It has many advantages, such as high accuracy, small volume, and light weight. Considering the integration level and capability of the device, we choose the supporting encoder for the selected Maxon motor as the angular sensor.

In addition, to improve the performance of this device, we have made some modifications. First, to reduce the overall mass, all linking bars are hollowed to proper thickness, and steel components are replaced by aluminum counterparts in light loading areas. Next, for a workspace free of singularities, arresting pins are applied to constrain extreme motions of the rhombus linkage. Balance weight blocks are added to eliminate the influence of gravity.

## 3. Motion Measurement and Tracking

**Figure 7:**
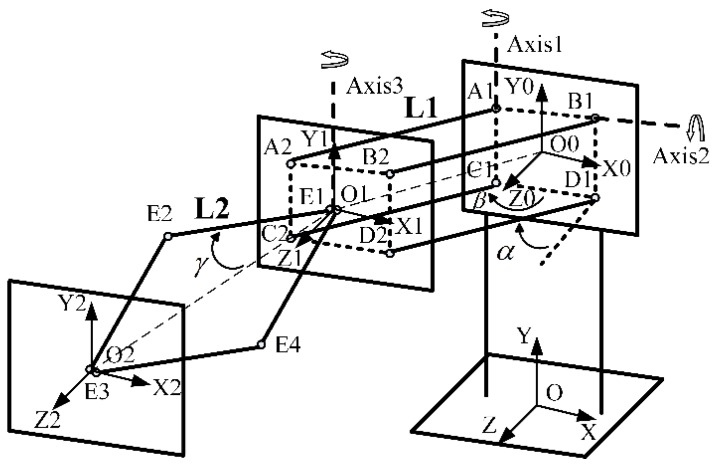
Motion measurement model of translational structure.

Motion measurement and tracking is one of the most fundamental tasks of a haptic control system. It is expected to generate real-time data that dynamically represents the pose changes of a human body (or a part of it) based on motion-sensing technologies. It needs kinematic data of motion sequences. As a result, positions of the end-effector and joint angles must be acquired. In the device, we use nine encoders to measure and track motions of the operator’s hand and fingers. The motion measurement model of the translational structure is shown in Figure 7.

For convenience of description, rotation angles of the double parallel linkage around axises 1 and 2 are defined as *β* and *α*. The angle between the linking bar E1 E2 and the centre line O1 O2 is set as *γ*. Lengths of the bar A1 A2 and E1 E2 are L1 and L2, respectively. The height between the point O0 and O is h. In the OXYZ coordinate system, the coordinate of point O0, O1 and O2 are ${\left(x_{0},y_{0},z_{0}\right)}^{T}$, $\;{\left(x_{1},y_{1},z_{1}\right)}^{T}$ and ${\left(x,y,z\right)}^{T}$, respectively. Therefore, the following equations are established.

(1) $${\left(x_{0},y_{0},z_{0}\right)}^{T}={\left(0,h,0\right)}^{T}$$

(2) $${\left(x_{1},y_{1},z_{1}\right)}^{T}={\left(x_{0},\;y_{0},z_{0}\right)}^{T}+\vec{O0O1}$$

(3) $${\left(x,y,z\right)}^{T}={\left(x_{1},y_{1},z_{1}\right)}^{T}+\vec{O1O2}$$

The vector $\vec{O1O2}$ is always perpendicular to the plane A2 B2 C2 D2, regardless of the location of the translational structure. Hence, the vector $\vec{O_{1}O_{2}}$ can be represented as:

(4) $$\vec{O1O2}={\left(0,\;0,L_{\text{O}1\text{O}2}\right)}^{T}$$

$L_{O_{1}O_{2}}$ is the length of the vector $\vec{O1O2}$.

(5) $$L_{O1O2}=2L_{2}\cos\gamma$$

Thus, the vector can be rewritten as:

(6) $$\vec{O1O2}={\left(0,\;0,\;2L_{2}\cos\gamma \right)}^{T}$$

The trajectory of the plane A2 B2 C2 D2 is a part of the spherical surface. Using the conversion between the spherical coordinate system and the Cartesian coordinate system, the vector $\vec{O0O1}$ is expressed as:

(7) $$\vec{O0O1}=\left[\begin{array}{c} \frac{L_{1}\cos\alpha \cos\beta }{\sqrt{{\left(\cos\alpha \right)}^{2}+{\left(\sin\alpha \sin\beta \right)}^{2}}}\\ \frac{L_{1}\sin\alpha \sin\beta }{\sqrt{{\left(\cos\alpha \right)}^{2}+{\left(\sin\alpha \sin\beta \right)}^{2}}}\\ \frac{L_{1}\cos\alpha \sin\beta }{\sqrt{{\left(\cos\alpha \right)}^{2}+{\left(\sin\alpha \sin\beta \right)}^{2}}} \end{array}\right]$$

Thus, the coordinate of the point O2 is acquired.

(8) $${\left(x,y,z\right)}^{T}=\left[\begin{array}{c} \frac{L_{1}\cos\alpha \cos\beta }{\sqrt{{\left(\cos\alpha \right)}^{2}+{\left(\sin\alpha \sin\beta \right)}^{2}}}\\ \frac{L_{1}\sin\alpha \sin\beta }{\sqrt{{\left(\cos\alpha \right)}^{2}+{\left(\sin\alpha \sin\beta \right)}^{2}}}+h\\ \frac{L_{1}\cos\alpha \sin\beta }{\sqrt{{\left(\cos\alpha \right)}^{2}+{\left(\sin\alpha \sin\beta \right)}^{2}}}+2L_{2}\cos\gamma \end{array}\right]$$

According to pervious equations, the coordinate of O1 is:

(9) $${\left(x_{1},y_{1},z_{1}\right)}^{T}={\left(x,y,z-2L_{2}\cos\gamma \right)}^{T}$$

The trajectory of the point O1 is a spherical arc. Therefore:

(10) $${\left(x_{1}-x_{0}\right)}^{2}+{\left(y_{1}-y_{0}\right)}^{2}+{\left(z_{1}-z_{0}\right)}^{2}={L_{1}}^{2}$$

Thus, three angles can be calculated:

(11) $$\{\begin{array}{c} \beta =\cos^{-1}\left(\frac{x}{\sqrt{L_{1}^{2}-{\left(y-h\right)}^{2}}}\right)\\ \alpha =\sin^{-1}\left(\frac{y-h}{\sqrt{L_{1}^{2}-x^{2}}}\right)\\ \gamma =\cos^{-1}\left(\frac{z-\sqrt{L_{1}^{2}-x^{2}-y^{2}}}{2L_{2}}\right) \end{array}$$

## 4. Realization of Force Feedback 

**Figure 8:**
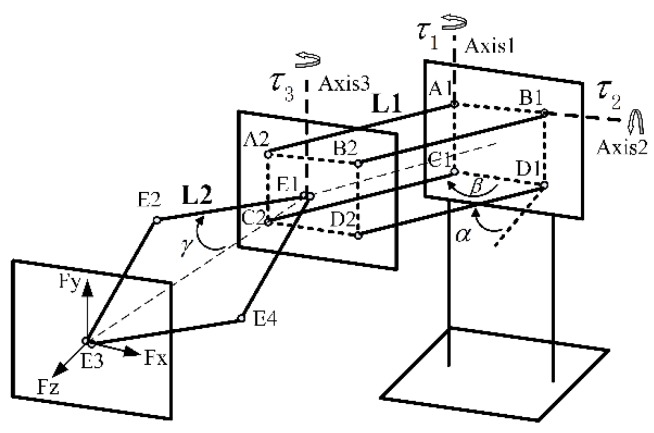
Model of translational force feedback.

Haptic-rendering algorithms compute interaction forces between avatars and objects when collisions are detected. Assume that outputs of three actuators along axises 1–3 are $\tau_{1}$, $\tau_{2}$ and $\tau_{3}$ respectively. $F_{x}$, $F_{y}$ and $F_{z}$ are components of force along three axes of the $O_{2}X_{2}Y_{2}Z_{2}$ coordinate system. The model of translational force feedback is illustrated in Figure 8. 

$F_{x}$ and $F_{y}$ can easily be acquired.

(12) $$\{\begin{array}{c} F_{x}=\frac{\tau_{1}}{d}\\ F_{y}=\frac{\tau_{2}}{d} \end{array}$$

d is the arm of force:

(13) $$d=\frac{L_{1}\cos\alpha \cos\beta }{\sqrt{{\left(\cos\alpha \right)}^{2}+{\left(\sin\alpha \sin\beta \right)}^{2}}}+2L_{2}\cos\gamma$$

The rhombus linkage has its own characteristics. The principle of virtual work [25,26] is adopted in the computation of $F_{z}$. The principle of virtual work states that the sum of the works of the internal and external forces done by virtual displacements is zero.

(14) $$-{\delta \text{W}}_{\text{internal }}-{\delta \text{W}}_{\text{external }}=0$$

**Figure 9:**
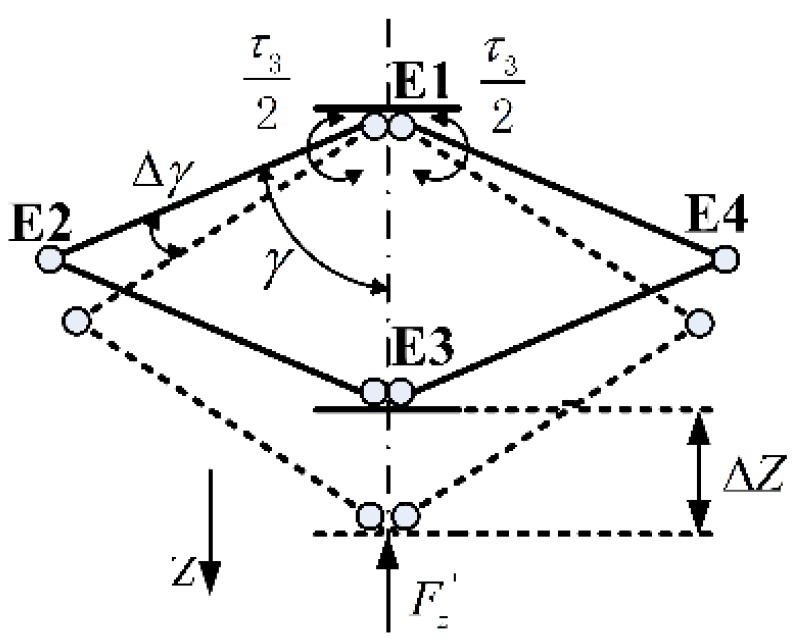
Model of virtual work.

Virtual displacements are infinitesimal changed in the position coordinates of a system such that the constraints remain satisfied. The model of virtual work is displayed in Figure 9.

∆Z is the infinitesimal change of the point E3. ∆γ is the tiny rotation angle of the linking bar E1-E2. $F_{z}^{'}$ represents the component of external force in the Z-axis. Therefore, the virtual work of the user’s hand $∆W_{1}$ and the actuator $∆W_{2}$ are computed as follows:

(15) $$\{\begin{array}{c} ∆W_{1}=-F_{z}^{'}\times ∆Z\\ ∆W_{2}=2\times \frac{\tau_{3}}{2}\times ∆\gamma \\ ∆W_{1}+∆W_{2}=0 \end{array}$$

**Figure 10:**
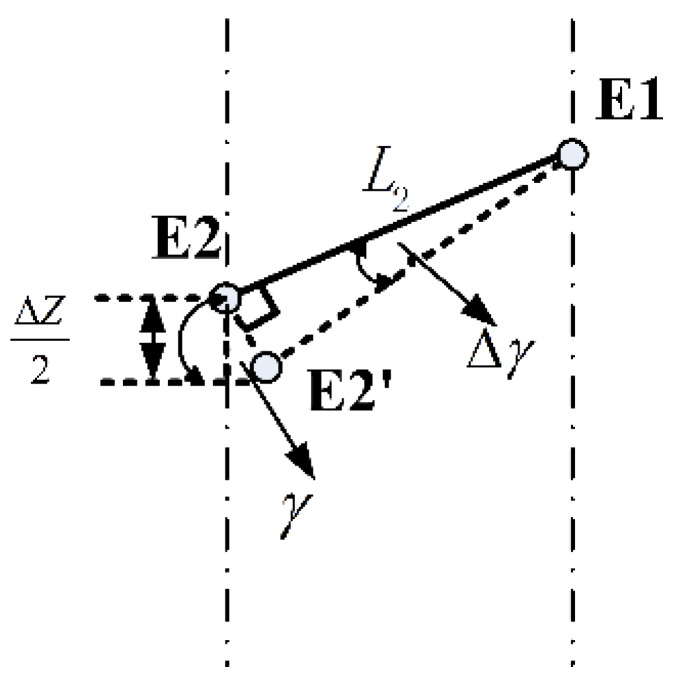
Approximation model.

Since ∆γ is small enough, the displacement E2 E2‘ can be treated as a vertical line of the linking bar E1 E2 (see Figure 10). Approximate equations are established:

(16) $$\{\begin{array}{c} \frac{∆Z}{2}=L_{E_{2}E_{2}^{'}}\times \sin\gamma \\ L_{E_{2}E_{2}^{'}}=L_{2}\times ∆\gamma \end{array}$$

Based on Equations (15) and (16), the force $F_{z}^{'}$ is calculated as:

(17) $$F_{z}^{'}=\frac{\tau_{3}}{2L_{2}\sin\gamma }$$

So $F_{x}$, $F_{y}$ and $F_{z}$ can be expressed as below:

(18) $$\{\begin{array}{c} F_{x}=\frac{\tau_{1}}{\frac{L_{1}\cos\alpha \cos\beta }{\sqrt{{\left(\cos\alpha \right)}^{2}+{\left(\sin\alpha \sin\beta \right)}^{2}}}+2L_{2}\cos\gamma }\\ F_{y}=\frac{\tau_{2}}{\frac{L_{1}\cos\alpha \cos\beta }{\sqrt{{\left(\cos\alpha \right)}^{2}+{\left(\sin\alpha \sin\beta \right)}^{2}}}+2L_{2}\cos\gamma }\\ F_{z}=\frac{\tau_{3}}{2L_{2}\sin\gamma } \end{array}$$

Shafts of the rotating mechanical structure are mutually perpendicular. $\tau_{4}$, $\tau_{5}$ and $\tau_{6}$ are independent torques in pitch, yaw and roll respectively.

(19) $$\{\begin{array}{c} M_{\text{pitch}}=\tau_{4}\\ M_{\text{yaw }}=\tau_{5}\\ M_{\text{roll }}=\tau_{6} \end{array}$$

Forces exerted on the three fingers are also independent. They are only relevant to the corresponding torques and arms. The motors in the grasping interface are numbered as NO. 7~NO. 9. Three grasping forces are shown as below:

(20) $$\{\begin{array}{c} F_{7}=\frac{\tau_{7}}{L_{7}}\\ F_{8}=\frac{\tau_{8}}{L_{8}}\\ F_{9}=\frac{\tau_{9}}{L_{9}} \end{array}$$

Thses forces are desired to be as close as possible to the forces that would arise during real-object contact. With accurate force feedback, operators were able to acquire an intuitive feeling about what they had touched. 

## 5. Control Scheme of the 6 DOF Haptic Device

**Figure 11:**
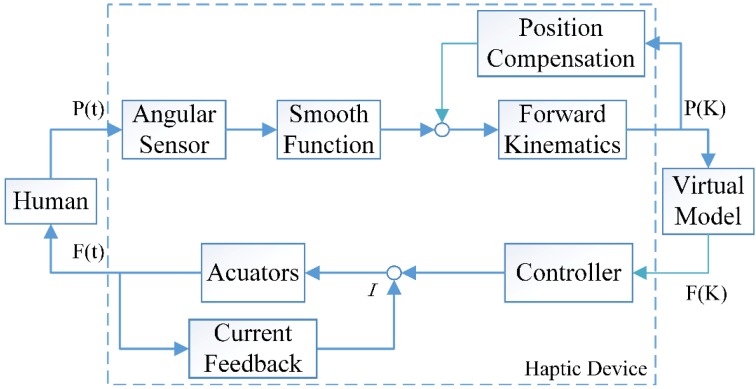
Control scheme diagram.

Haptic interactions require both motion collection and actuator control. The control scheme of this haptic device is illustrated in Figure 11. Motions of the operators’ hand and fingers are collected by encoders. Through the smooth function, the data is transmitted to forward kinematic equations for position and orientation calculation. Position information is compensated before sent to the haptic rendering engine for collision detection. Interaction forces are computed by modeling a spring between the avatar and the device.

(21) $$F\left(x\right)=\{\begin{array}{cc} -kx, & if\;x>0\\ 0, & otherwise \end{array}$$

k represents the object’s stiffness. Force commands are transferred back to generate corresponding forces under the condition that the current of all motors are less than the peak values. In the diagram, P(t) and F(t) are continuous-time position and force signals exchanged between the human user and the haptic device. P(K) and F(K) are discrete-time position and force signals exchanged between haptic device and virtual environment. 

To ensure the accuracy of computed positions and orientations at a high level, a smoothing function is used to filter peak values that may cause disturbances. It acts as follows. First, three successive frames of position data are received and saved. Let $P_{previous}^{i}$ be the position data of motor NO.*i* received at the previous frame, let $P_{current}^{i}$ be the position data at the current frame, and let $P_{next}^{i}$ be the position data at the next frame. $P_{error}^{+}$ and $P_{error}^{-}$ represent the maximum positive position error and negative position error, respectively. Without loss of generality, we assume that the three frames of data all meet the communication protocols and there is no data loss. Three frames of data is subsequently checked. The smoothing function is described in Algorithm 1.

**Algorithm 1** Peak Values Filter of Current Frame of Data
$function\text{ Smoothing Function }\left(P_{previous}^{i},\;P_{current}^{i},\;P_{next}^{i}\right)$
 for i=0 →8 do      $if(P_{current}^{i}-P_{previous}^{i}>P_{error}^{+}\&\&P_{current}^{i}-P_{next}^{i}>P_{error}^{+})\;$      $discard\;\;P_{current}^{i}$    $else\;if\;(P_{current}^{i}-P_{previous}^{i}<P_{error}^{-}\&\&P_{current}^{i}-P_{next}^{i}<P_{error}^{-})$      $discard\;\;P_{current}^{i}$    end if end for $return\;\;P_{current}^{i}$end function

There are nine motors and encoders in the haptic device. In order to maintain a stable system while displaying smooth and realistic forces and torques, the force control rate must be as high as possible. As described above, the actuator chosen was the Maxon DC motor, which has a short response time of about 2~3 ms. Force commands were controlled at the frequency of 300 HZ. 

## 6. Calibrations and Evaluations

### 6.1. Assessment of Position Tracking Accuracy

**Figure 12:**
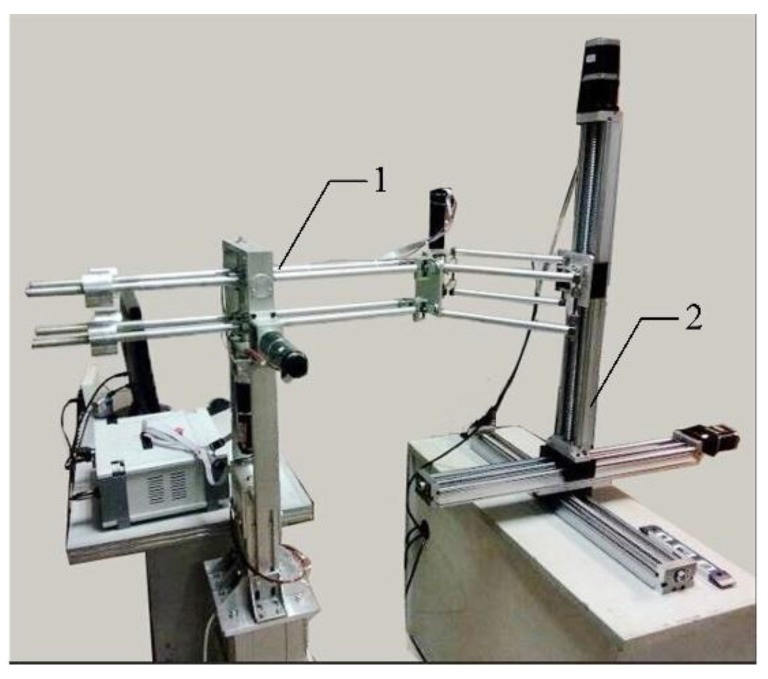
Setup of position tracking experiment (**1**) Haptic device; (**2**) Three dimensional electric sliding platform.

Position tracking experiments are conducted on a three dimensional electric sliding platform that has a linear precision of 5/57 mm and an effective stroke of 600 mm on each axis (see Figure 12). The platform is driven by three step-motors with two operating modes. During tests, we use the manual mode to provide 2 mm displacement at each step. To protect the device from running out of strokes and to reduce repeated measurements, we adopted the following strategies: first, the workspace was measured in advance; afterward, the device was allowed to track the sliding platform step by step within a given range for specific assessment. Actual displacements (displacements of the sliding platform) can be read from the LCD (Liquid Crystal Display) panel of the platform control box. Computed displacements (displacements of the haptic device) are displayed in the window of Force Feedback Controller, a software programmed for this haptic device. 

Measurements show that the workspace of the X-axis, Y-axis and Z-axis reach 500 mm, 500 mm and 420 mm, respectively. This is a comparatively large workspace when compared to other haptic devices. The most commonly used PHANTOM series devices provide workspaces of different sizes. PHANTOM Omni only provides 160 × 120 × 70 mm in width, height and depth. PHANTOM Premium 1.5 provides a larger workspace of 381 × 267 × 191 mm, but its workspace is still smaller than ours. PHANTOM Premium 3.0 can provide a range of motion that is equivalent to human shoulder rotation arm movement. Its workspace reaches 838 × 584 × 406 mm, but our device can offer a larger range of movement in the Z direction. Another family of haptic devices is manufactured by Force Dimension. The Force Dimension devices utilize parallel kinematic design and provide small workspaces. The Delta series offers a slightly larger workspace of ϕ 400 × 260 mm, which is still smaller than the workspace of our device. The workspace of Sigma 7, the most advanced haptic device ever designed by Force Dimension, is even smaller. Another commercially available device, HD^2^ High Definition, has a workspace of 800 × 250 × 350 mm. Out of these three dimensions, two of them are less than what we have [27].

**Figure 13:**
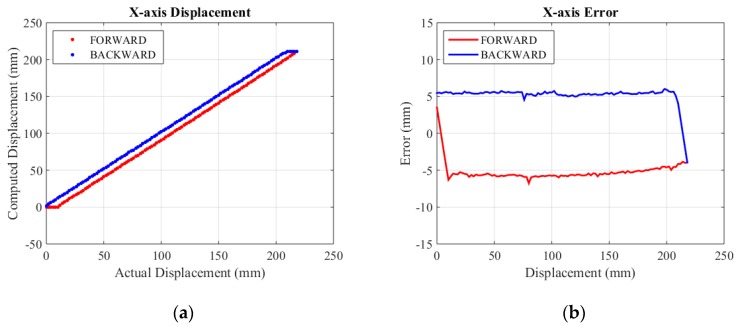
X-axis displacement and error. (**a**) Displacement; (**b**) Error.

**Figure 14:**
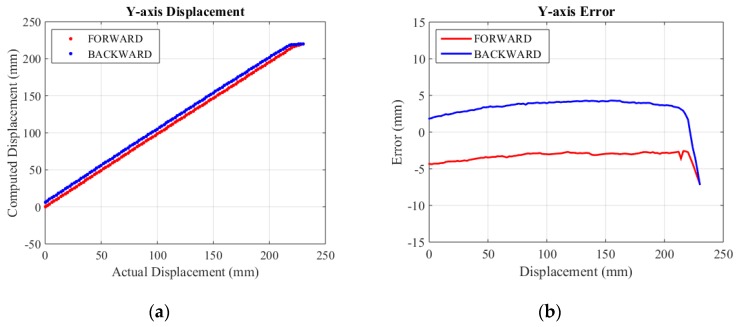
Y-axis displacement and error. (**a**) Displacement; (**b**) Error.

**Figure 15:**
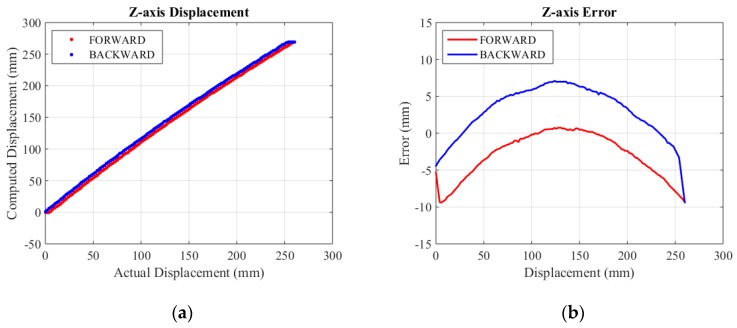
Z-axis displacement and error. (**a**) Displacement; (**b**) Error.

The actual specific assessment range of X-axis, Y-axis and Z-axis are selected as 0~218 mm, 0~230 mm and 0~260 mm. Experimental results are shown in Figure 13, Figure 14 and Figure 15, respectively.

For the convenience of analysis, we defined computed error as the difference between mechanical error and displacement error. Displacement error mainly comes from mechanical error and computed error. The mechanical error stems from small backlashes (mainly gear clearance) in the structure. Linking bars magnify these backlashes and make errors relatively large. From the initial error figures above, it can be seen that the mechanical error of the X-axis is about 10 mm, while the mechanical error of the Y-axis is smaller than 10 mm because of the effect of gravity. The error figure of the Z-axis is a curve, because its displacement is relevant to cosγ (shown in Equation (8)). The mechanical error of the Z-axis is the smallest, since linking bars of the rhombus linkage are shorter than those of the double parallel linkage. The computed error lies in inaccuracy of the algorithm and a limited resolution of encoders. The computed error is estimated by its MSE (mean square error). The initial MSE of computed errors in the forward and backward travel in X-axis, Y-axis and Z-axis are calculated to be 0.37 mm and 0.24 mm, 0.65 mm and 0.52 mm, 3.14 mm and 3.53 mm, respectively. Compared to mechanical error, computed error contributes to only a small portion of the overall displacement error.

**Figure 16:**
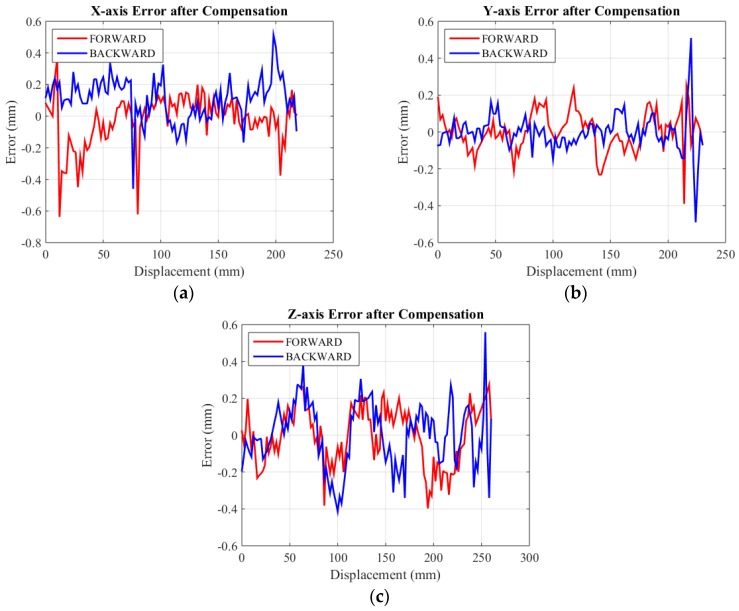
Displacement error after compensation of three axes. (**a**) Displacement error of X-axis; (**b**) Displacement error of Y-axis; (**c**) Displacement error of Z-axis.

After the analysis of displacement error, we made some modifications on the algorithm to compensate for mechanical error and to reduce computed error. Due to the dramatic differences of displacement error at the beginning and end of travels along the three axes, segmented error compensation is utilized. Compensated data of three axes are displayed in Figure 16. The standard deviation of the mean values of the displacement errors in forward and backward travel along the three axes are calculated to be 0.1591 mm and 0.1329 mm, 0.1051 mm and 0.1053 mm, 0.1572 mm and 0.1742 mm after compensation. The maximum displacement errors are 0.63 mm, 0.51 mm and 0.56 mm along X-axis, Y-axis and Z axis. 

### 6.2. Calibration of Static Output Forces 

**Figure 17:**
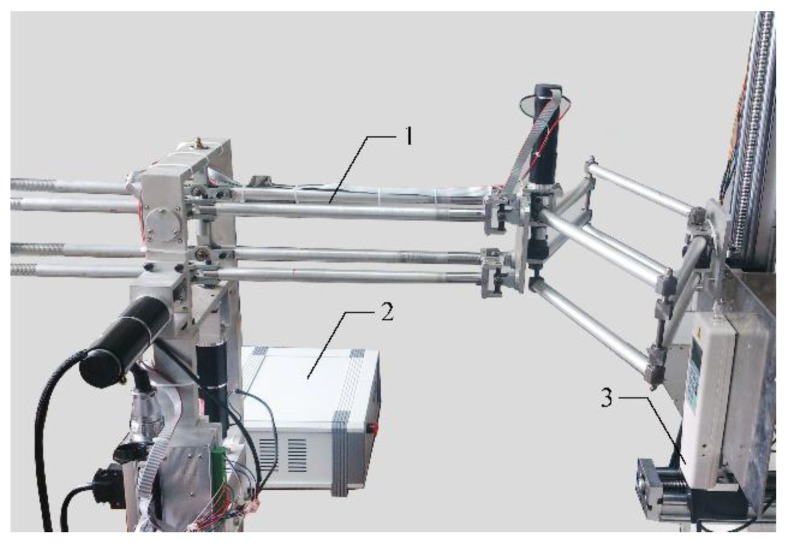
Setup of static translational force calibration (**1**) Haptic device; (**2**) Haptic control box; (**3**) Digital force gauge.

For a haptic device, small forces are insufficient to construct a high level of immersion, while ultra-strong forces may hurt the manipulator and damage device. Thus, it is important to have desirable output. Static output forces of this device is measured by a digital force gauge, which has a peak force of 30 N and a force resolution of 0.01 N (shown in Figure 17). The gauge is equipped with a serial port through which sampled data can be stored automatically. In experiments, peak forces were measured first. Subsequently, static output forces were specifically calibrated within continuous working ranges. 

**Figure 18:**
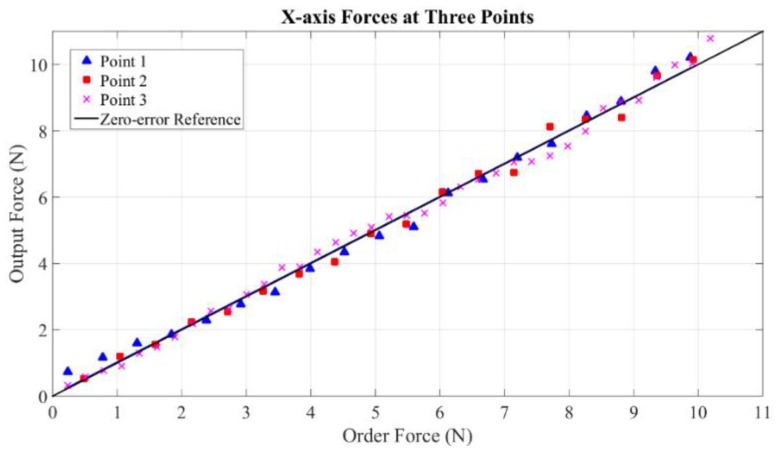
Forces at three points along X-axis.

**Figure 19:**
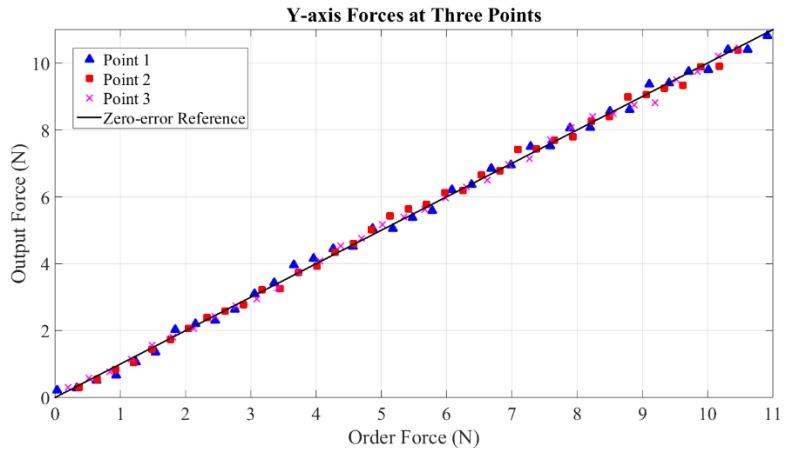
Forces at three points along Y-axis.

**Figure 20:**
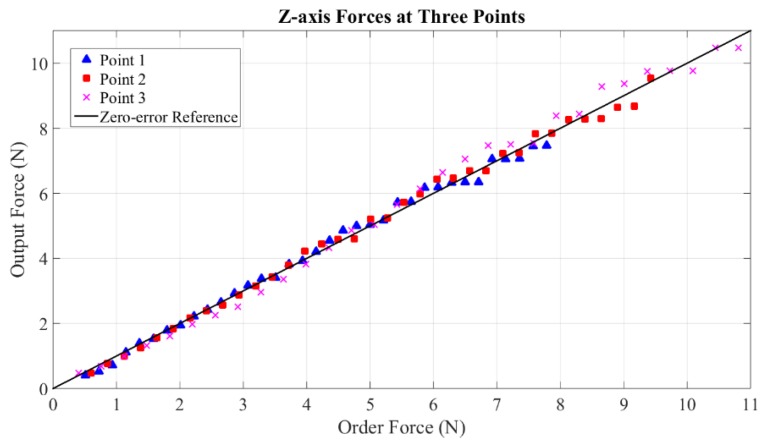
Forces at three points along Z-axis.

Maximum measurable forces of X-axis, Y-axis and Z-axis are 20 N, 20 N and 19 N, respectively. These values reach the level of maximum force output of PHANTOM Premium 3.0 (22 N), Deltas 3 and 6 (20 N), and Sigma 7 (20 N). Without loss of generality, static forces are specifically calibrated at five points within continuous working ranges along the three axes. Three representative experimental results of each axis are shown in Figure 18, Figure 19 and Figure 20. From the results, forces of the three points along the X-axis are displayed with average error and maximum error of 0.0205 N and 0.48 N, 0.000384 N and 0.41 N, 0.0084 N and 0.46 N. The errors of the three points along the Y-axis are even smaller with the largest average error of 0.000233 N and largest maximum error of 0.38 N. Similarly, the largest average error of the three points along the Z-axis are computed as 0.0112 N and the largest maximum error is 0.49 N. 

**Figure 21:**
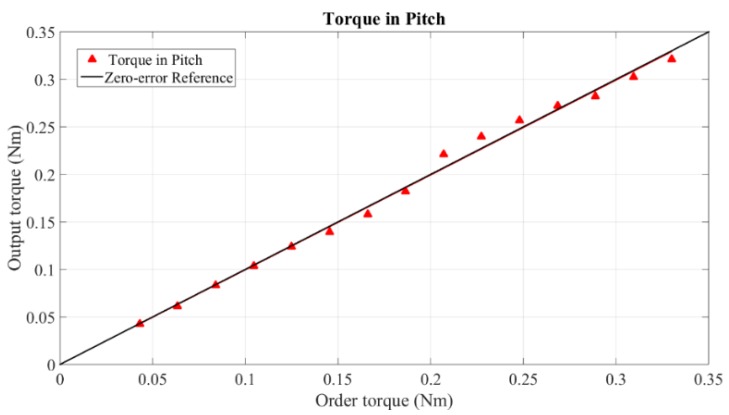
Torque in pitch.

**Figure 22:**
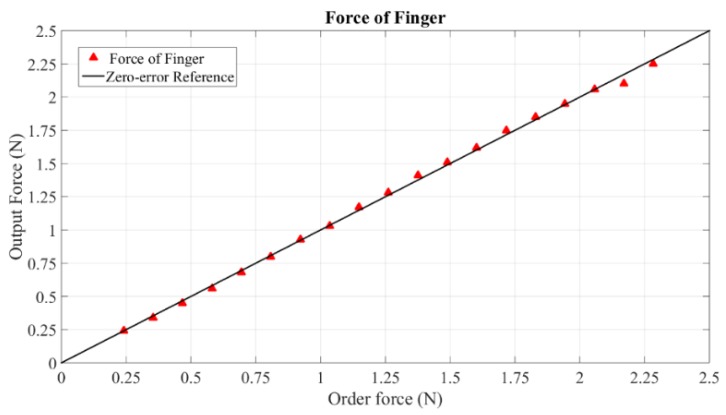
Force of grasping interface.

Since the digital force gauge is unable to measure torques, torque calibration is based on force measurement with the given arm of 165 mm. Representative experimental results (torque in pitch and force of middle finger) are shown in Figure 21 and Figure 22. The measurable peak torque is 0.6 Nm. This value is larger than 0.15 Nm of Delta 6 and 0.4 Nm of Sigma 7, but less than 1.72 Nm of HD^2^ High Definition. From the data of Figure 21, the average error of torque is 0.00049 Nm with a maximum error of 0.0087 Nm. The measurable peak force of our grasping interface reaches 3.5 N, which is less than the maximum grasping force of 8 N provided by Sigma 7. However, our grasping interface provides more accurate forces. The maximum error of grasping force is 0.07 N with the average error only 0.0001 N. The specific error data is given in Table 1.

**Table 1:** Errors of Force/Torque.

Force/Torque	Group	Average Error	Maximum Error
Forces in X-axis	Point1	0.0205 N	0.48 N
	Point2	0.000384 N	0.41 N
	Point3	0.0084 N	0.46 N
Forces in Y-axis	Point1	0.000315 N	0.27 N
	Point2	0.000162 N	0.27 N
	Point3	0.000233 N	0.38 N
Forces in Z-axis	Point1	0.000571 N	0.36 N
	Point2	0.000285 N	0.49 N
	Point3	0.0112 N	0.45 N
Torque	Pitch	0.00049 Nm	0.0087 Nm
Grasping interface	Middle finger	0.0001 N	0.07 N

Since the forces measured are static, friction effects should be evaluated to improve the transparency of the device. Static-friction force is measured by gradually increasing the input value from zero. The minimum measurable force and the corresponding input value are recorded. From the known relation between input value and output force, the static-friction force can be calculated. The static-friction force of X-axis, Y-axis and Z-axis are 0.32 N, 0.30 N and 0.40 N, respectively. 

After evaluations, specific properties of this haptic device are displayed in Table 2.

**Table 2:** Device Properties.

Properties	Values
Workspace	Translation: 500 × 500 × 420 mm in X-,Y- and Z-axis
	Rotation: pitch −35°~90°, roll ±60° and yaw ±90°
	Grasping interface: 5 cm
Position resolution	0.01 mm
Peak output ability	Translation: 20 N in X-, Y-axis and 19 N in Z-axis
	Rotation: 0.6 Nm
	Grasping interface: 3.5 N
Continuous working range	Translation: 10 N in X-, Y- and Z-axis
	Rotation: 0.3 Nm
	Grasping interface: 2 N
Force resolution	0.01 N

### 6.3. Application Examples 

**Figure 23:**
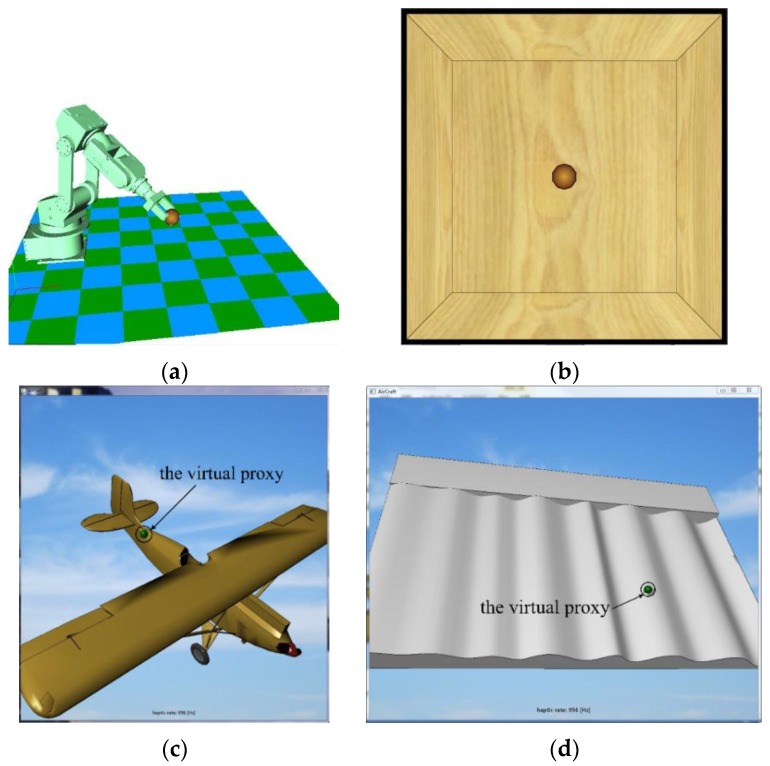
Four application examples: (**a**) Grasping example; (**b**) Bounding box example; (**c**) Rigid object interaction; (**d**) Position tracking example.

Four application examples (shown in Figure 23) are programmed to identify the capability and potential of the device. The first example is designed to show the potential of the grasping interface. Operators are asked to operate the robot arm to grasp the ball in the virtual environment. When the ball is grasped, it follows the movement of the robot arm extremity, and forces are exerted on three fingers. A virtual bounding box with a size of 500 × 500 × 400 mm, is created to verify the effective workspace of this device. The virtual proxy [28] (similar to the “god-object” [29]) through which users physically interact with the virtual environment is modeled as a ball. Operators can move the ball freely inside the workspace. Once collisions between the ball, and boundaries of the box are detected, the motion of the ball is constrained immediately. During operations, we find that boundary of the box can be easily reached. The last two examples are programmed based on the CHAI-3D platform, with which designers can program their own demos easily. The third demonstration is designed to test the maximum force of our device. A yellow rigid aircraft is imported and fixed in the haptic scene. Operators are asked to hit the aircraft surface with the ball in different directions. At the same time, interaction forces are recorded. Interaction data shows that the maximum recorded forces of X-axis, Y-axis and Z-axis reach 20 N, 20 N and 19 N, respectively. The last demonstration is designed to verify the position tracking accuracy. A plate with varying waviness is created. Wave amplitude gradually increases from left to right. The ball is controlled to slide along the plate surface. From the users’ perspective, they can identify the surface feature of the plate. Moreover, they can distinguish force differences caused by tiny changes in wave amplitude.

## 7. Conclusions

In this study, a new 6 DOF hybrid structure haptic device was designed and calibrated. The device is mainly composed of a double parallel linkage, a rhombus linkage, a rotating mechanical structure and a grasping interface. It can provide three DOF of force feedback, three DOF of torque feedback and three independent grasping force feedback to human operators. Experiments were conducted on a three-dimensional electric sliding platform to evaluate the position tracking accuracy of this device. After analysis of displacement error, we made some modifications on the algorithm to compensate for mechanical error and to reduce computed error. Static output forces were calibrated by a digital force gauge. Friction effects were evaluated to improve the transparency of the whole system. To show the capability and potential of our device, four application examples were programmed. Calibration and evaluation results show that this is an efficient haptic device with a large workspace and a high output ability.
